# Defining the hemodynamic response of hypertensive and normotensive subjects through serial timed blood pressure readings in the clinic

**DOI:** 10.1186/s40885-019-0114-z

**Published:** 2019-04-01

**Authors:** Hunaina Shahab, Hamza Sohail Khan, Aysha Almas, Sohail Abrar Khan, Azmina Artani, Aamir Hameed Khan

**Affiliations:** 1 Aga Khan University, P.O. Box 3500, Stadium Road, Karachi, 74800 Pakistan; 2 Tabba Heart Institute, St-1, Block 2, Federal B Area, Karachi, 75950 Pakistan

**Keywords:** White-coat effect, White-coat hypertension, Post-clinic blood pressure

## Abstract

**Background:**

Every third patient in the clinic is misdiagnosed due to white-coat phenomenon, necessitating needless and costly treatment. We aimed to study the hemodynamic response of the physician’s visit on hypertensive and normotensive patients by investigating the trend of blood pressure (BP) before, during and 15 min after the physician-patient encounter.

**Methods:**

A descriptive, cross-sectional study was conducted over a period of 8 months in the cardiology clinics at the Aga Khan University Hospital, Karachi. Both hypertensive and normotensive patients, aged ≥18 years, were recruited. Pregnant females or those with a history of volume loss were excluded. BP readings were taken using an automated, validated device (Omron-HEM7221-E) at three points: pre-clinic BP by the assessment nurse, in-clinic BP by the attending physician and post-clinic BP 15-min after the physician-patient encounter by a research assistant. Independent samples t-test was used to calculate the statistical difference between hypertensive and normotensive BP values.

**Results:**

Of 180 participants, 71% (*n* = 128) were hypertensive and 57% (*n* = 103) of all were males. The mean age of the participants was 57 ± 15 years. The mean and standard deviation(±SD) systolic BP (SBP) taken pre-clinic, in-clinic and 15-min post-clinic for hypertensive population was 128.7 ± 20 mmHg, 137.1 ± 21 mmHg and 127.9 ± 19 mmHg. The mean and standard deviation(±SD) SBP taken pre-clinic, in-clinic and 15 min post-clinic for normotensive population was 112 ± 16 mmHg, 115.8 ± 20 mmHg and 111.8 ± 15 mmHg. The hypertensive SBP values showed statistically significant difference from the normotensive values (difference in pre-clinic SBP: 16.7 mmHg, *p*-value < 0.001; in-clinic SBP: 21.3 mmHg, p-value < 0.001; and 15 min post-clinic: 16.1 mmHg, p-value < 0.001).

**Conclusions:**

Hypertensive and normotensive patients display congruent hemodynamics upon visiting the physician, the alert response being accentuated amongst the hypertensive group. In-clinic BP readings are higher for both hypertensive and normotensive patients making them unreliable for screening and management of hypertension amongst both the groups.

## Background

Hypertension is a coronary risk factor which poses significant health challenge around the globe, most of the disease burden caused by high blood pressure (BP) being borne by lower and middle-income countries [1]. The prevalence of hypertension in Pakistan is reported to be around 34% [2]. This accounts for 25% of all deaths in the country [3]. The ongoing Control of Blood Pressure and Risk Assessment trial (COBRA-BPS), being conducted in three South Asian countries, has recently reported that an alarming proportion of hypertensive patients in Pakistan (nearly 71%) have uncontrolled BP [4]. Hence, the accuracy in measurement and routine follow-up of these patients becomes immensely important.

Office blood pressure (BP) is unable to overcome the white-coat effect [5], which affects up to 33% of patients [6, 7]. The Post Clinic Ambulatory Blood Pressure (PC-ABP) study conducted by our research group showed that the prevalence of white-coat effect in Pakistan was about 38% and about 25% of the patients had white-coat hypertension [8]. This implies that about one in three to four patients are misdiagnosed in the office setting leading to the prescription of needless and costly antihypertensive medications [9]. We previously conducted the post-clinic blood pressure (PC BP) study to show the trend of BP readings amongst all patients visiting an outpatient cardiology clinic, in an attempt to investigate which BP reading was the lowest amongst all taken in a routine clinic visit [10]. We now aimed to determine the hemodynamic difference amongst hypertensive and normotensive patients on meeting the physician by studying the BP and pulse before, during and 15 min after the physician-patient encounter to determine the alert response when the patient visits the physician in the clinic. To complete our objective, we conducted a sub-analysis of the post-clinic BP study [10].

## Methods

### Study site, population and sample size

The methodology of this study is already published and available in our previous paper on post clinic BP [10]. In brief, it was a cross-sectional, descriptive study conducted over a period of 8 months in the outpatient cardiology clinics at Aga Khan University Hospital, Karachi, Pakistan. Patients of ≥18 years of age, who were either hypertensive (defined as those with a clinic SBP ≥140 mmHg or DBP ≥90 mmHg [11]) or normotensive were recruited. The normotensive participants were those patients who were visiting the cardiology clinics for indications other than hypertension like chronic stable angina, dyslipidemia, follow-up post percutaneous intervention (PCI) or post coronary artery bypass grafting (CABG) etc. Participants who had a history of diarrhea or any other kind of volume loss, pregnant females, those taking non-steroidal anti-inflammatory drugs or those who had taken an extra dose of anti-hypertensive medication before the clinic visit were excluded.

A sample size of 101 participants was needed to calculate a mean difference among clinic readings of 7.5 mmHg systolic BP (SBP) and 2.9 mm diastolic BP (DBP) at an alpha of 5% and a beta of 80%. After accounting for a 10% dropout rate, the minimum sample size needed to show the differences in readings taken at various points within the clinic was 110. A total of 180 participants were enrolled in the study.

### Blood pressure and pulse measurements

Blood pressure and pulse readings were taken at three different points in the clinic. The first set of pre-clinic readings were taken by the assessment nurse 16 ± 1.7 min after the patient had entered the hospital. The next set of in-clinic readings were taken by the physician 15 ± 2.1 min after the pre-clinic readings. Then the participants were asked to wait for another 15 min in the regular waiting area where they were asked to refrain from smoking and/ or exertion. Our rationale for waiting 15 min before taking the post-clinic readings was based on van der Wel et al’s study which showed that BP readings reach a plateau over 15 min [12]. This was also done to match the unavoidable waiting time that each patient is subjected to before getting seen by the pre-clinic assessment nurse and the attending physician. The last set of post-clinic readings were taken after 15 ± 1.3 min in another clinic room by a research assistant. At each point, 2 sets of BP and pulse readings were taken with an interval of 1 min in between. In the current study, we decided to use the average of the post-clinic readings in the final analysis.

At the start of the study, all three observers taking the readings in the pre-clinic, in-clinic and post-clinic periods were given a small training course on the proper method of measuring blood pressure to ensure standardization. The method used was as follows: each participant sat on a chair with the back supported and feet placed flat on level ground; arm was supported at heart’s level; an adequate size cuff was used, the bladder of which covered 80% of the arm [11]; with an automated and validated BP device [13] (Omron HEM 7221-E, M6 Comfort, Omron Healthcare Europe). This was done to reduce inter-observer variability in the readings. Talking was not allowed during the measurement of readings.

### Statistical analysis

We used the Statistical Package for Social Sciences (SPSS), version 23 for re-analyzing our data. For quantitative variables were used mean and standard deviation (SD) whereas for qualitative variables were used frequency and percentages. A mean was calculated on the basis of all the readings measured during different points in the clinic. Independent samples t- test was used to determine the statistical difference between hypertensive and normotensive SBP, DBP and pulse values. We also determined the trend of BP among those using anti-hypertensive and compared them with those not using anti-hypertensive medications at each point in the clinic by employing t-test for independent sample.

## Results

A total of 200 patients were contacted to participate in the study. Of the total, due to lack of time on part of the participants, 12 (6%) refused to enroll themselves in the study whereas 8 (4%) left before the 15 min waiting period was over. Therefore, the total number of participants was 180. The mean age of the participants was 57 ± 15 years. Of the 180 participants, 57% (*n* = 102) were males. Of all participants, 71% (*n* = 128) were hypertensive of which 92% patients were on antihypertensive medications. Baseline characteristics of our study population is shown in Table 1.

**Table 1:** Baseline characteristics comparison between hypertensive and normotensive patients (*n* = 180)

Variables	Normotensive patients (*n* = 52)	Hypertensive patients (*n* = 128)	P-value for Difference
Mean Age in years (±SD)	48.5 (16.8)	60.9 (11.3)	< 0.001
Mean BMI (±SD)	26 (5.9)	28.4 (5.2)	0.03
Female gender (n, %)	22 (42.3)	55 (43.0)	0.9
Diabetes (n, %)	5 (9.6)	42 (33.0)	0.001
Dyslipidemia (n, %)	12 (23.1)	46 (35.9)	0.09
Ischemic heart disease (n, %)	5 (9.6)	29 (22.6)	0.04
Chronic kidney disease, Stage 2 (n, %)	0	2 (1.6)	0.36
Paroxysmal Atrial fibrillation	4 (7.7)	7 (5.5)	0.57
Supraventricular Tachycardia (n, %)	1 (1.9)	0	0.11
Thyroid disorders (n, %)	2 (3.8)	1 (0.8)	0.15
Valvular heart disease	3 (5.8)	4 (3.1)	0.4
Cardiomyopathy	1 (1.9)	0	0.11
History of PCI or CABG (n, %)	3 (5.8)	20 (15.6)	0.07

Abbreviations, *PCI* Percutaneous Coronary Intervention, *CABG* Coronary Artery Bypass Grafting, *SD* Standard Deviation

**Fig. 1:**
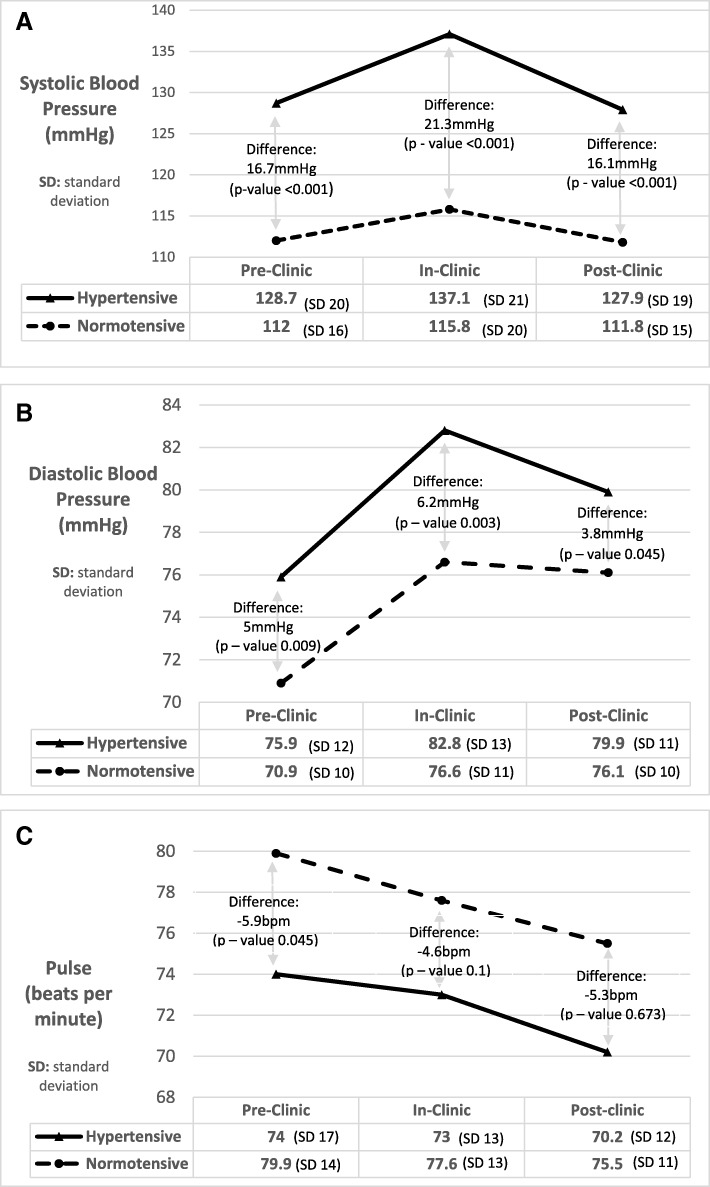
**a** Trends in Systolic Blood Pressure in mmHg amongst Hypertensive and Normotensive Patients. Figure shows an increase in systolic blood pressure from pre-clinic time to in-clinic period followed by a drop in the post-clinic phase. Arrows denote the difference between hypertensive and normotensive systolic blood pressure in the pre-clinic, in-clinic and post-clinic phase along with their *p*-values. **b** Trends in Diastolic Blood Pressure in mmHg amongst Hypertensive and Normotensive Patients. Figure shows an increase in diastolic blood pressure from pre-clinic time to in-clinic period followed by a drop in the post-clinic phase. Arrows denote the difference between hypertensive and normotensive diastolic blood pressure in the pre-clinic, in-clinic and post-clinic phase along with their p-values. **c** Trends in Pulse in beats per minute amongst Hypertensive and Normotensive Patients. Figure shows a consistent decrease in pulse in beats per minute from pre-clinic to post-clinic phase. Arrows denote the difference between hypertensive and normotensive pulse values in the pre-clinic, in-clinic and post-clinic phase along with their *p*-values

The mean and standard deviation (±SD) systolic blood pressure (SBP in mmHg) taken pre-clinic, in-clinic and 15 min post-clinic amongst hypertensive and normotensive participants is shown in Figure-1A. The mean and standard deviation (±SD) diastolic blood pressure (DBP in mmHg) taken pre-clinic, in-clinic and 15 min post-clinic amongst hypertensive and normotensive participants is shown in Figure-1B. The mean and standard deviation (±SD) pulse (in beats per minute) taken pre-clinic, in-clinic and 15 min post-clinic amongst hypertensive and normotensive participants is shown in Figure-1C. The difference between BP values in hypertensive and normotensive groups during the pre-clinic, in-clinic and post-clinic phase are shown on the respective Fig. 1a, b and c as denoted by arrows.

The difference between BP values in hypertensive patients (*n* = 128) on antihypertensive medications versus those who were not on antihypertensive medications is shown in Table 2. The 10 hypertensive patients who were not on antihypertensive medications were well-controlled on diet and lifestyle modifications. Both the antihypertensive user group and nonuser group display an increase in SBP and DBP upon visiting the physician and subsequent decrease in the post-clinic phase.

**Table 2:** Difference in pre-clinic, in-clinic and post-clinic blood pressure among hypertensive patients (n = 128) who were users (n = 118) versus non-users (n = 10) of anti-hypertensive medications

Variables	Anti-hypertensive non-users (*n* = 10)	Anti-hypertensive Users (*n* = 118)	*P*-value for Difference
Systolic Blood Pressure (measured in mmHg)			
Pre-Clinic Systolic (Mean, SD)	125.5 (5.9)	129.0 (1.8)	0.59
In-Clinic Systolic (Mean, SD)	133.6 (7.0)	137.4 (2.0)	0.61
Post-Clinic Systolic (Mean, SD)	123.8 (5.4)	130.7 (1.8)	0.25
Diastolic Blood Pressure (measured in mmHg)			
Pre-Clinic Diastolic (Mean, SD)	80.4 (4.2)	75.5 (1.1)	0.28
In-Clinic Diastolic (Mean, SD)	86.2 (4.4)	82.5 (1.8)	0.43
Post-Clinic Diastolic (Mean, SD)	82.5 (4.0)	79.7 (1.1)	0.52

## Discussion

In the post-clinic BP study, we showed that the BP in the post-clinic period is about 10 mmHg lower than the BP readings taken in the presence of the physician [10]. The next PC-ABP (post clinic ambulatory blood pressure) study showed that this post-clinic BP reading correlated well with 24 h overall ambulatory BP mean and the daytime ambulatory BP mean, the correlation being stronger for systolic readings, based on which we recommended the use of post-clinic BP for the management of hypertension in the clinic setting [8]. The PC-ABP study also showed the same trend of BP readings amongst the patients visiting the clinic hence the pattern is reproducible [8]. We now showed that the trends of BP in the clinic were congruent for both hypertensive and normotensive patients. However, the BP response shown by hypertensive patients was accentuated as compared to the normotensive. This is similar to the Ogedegbe et al’s study which investigated the anxiety scores and BP trends amongst sustained hypertensive, white-coat hypertensive, masked hypertensive and normotensive patients [14]. Our hypertensive population follows the same pattern as the white-coat hypertensive group investigated by Ogedegbe et al., whereby they raise their BP upon physician’s entrance to the clinic room and subsequently drop their BP readings after the physician’s exit [14]. However, a contrast from their study is amongst the normotensive participants. Our normotensive patients’ BP parallels the hypertensive group but their BP rise during the clinic visit is only modest. As opposed to this, the normotensive patients in Ogedegbe et al’s study experience a fall in the BP readings in the physician’s presence [14]. Furthermore, a case-control study conducted by Stergiou et al. [15] demonstrated that white-coat effect was still present in treated hypertensive patients compared to the untreated patients, albeit reduced. In our study both the groups of hypertensive patients (antihypertensive medications users and nonusers) display an increase in BP on visiting the physician followed by a drop in the post-clinic phase.

As we did not study the anxiety scores of the patients participating in our study, our results may not be able to determine the correlation of anxiety level with the BP readings taken at each point in the clinic. However, we believe that the accentuated rise in BP in the physician’s presence amongst the hypertensive patients is due to the greater effect of alert response of the clinic visit amongst these patients and this is similar to Spruill et al’s study where they showed that the patients’ perception of being hypertensive confers a greater state anxiety score and therefore white-coat effect [9]. Similarly, Pickering et al. identified that almost all patients with hypertension had at least some level of white-coat effect [16].

There are two theories to explain the white-coat phenomenon. The generalized anxiety theory states that the patients with a general tendency of getting anxious display white-coat hypertension [17]. The second theory of classical conditioning suggests that white-coat hypertensive patients are those who have previously been exposed to disagreeable stimuli like a poor diagnosis or a painful procedure in the clinic room, i.e. an unconditioned stimulus which, when paired with conditioned stimuli like the physician’s white coat or the office environment, leads to anxiety and therefore elevated BP [14, 18]. Although our results might not be able to prove a correlation, they do lend support to the classical conditioning theory whereby the stimulus of meeting the physician increase both hypertensive and normotensive patients’ BP readings which later decreases once the stress factor of meeting the physician is over. In all the three points in the clinic where BP and pulse were measured, the clinic room/ unconditioned stimuli in the environment remains the same. In such a scenario, the maximum rise in the BP occurs in the presence of the physician thereby we can hypothesize that the alert response is shown due to the physicians presence.

## Conclusion

Both hypertensive and normotensive patients show congruent hemodynamic trends during a physician-patient encounter, the alert response being accentuated in the diagnosed hypertensive. In-clinic BP readings are higher for both hypertensive and normotensive patients, therefore, they are not reliable to screen or manage hypertension in both the groups.

## Limitations

- It is possible that different antihypertensive medications may modify the BP response shown by the participants which was not studied due to a limited sample size.
- The level of anxiety among our study participants were not studied therefore we were not able to correlate BP readings with anxiety scores.
- The readings were taken by three different observers as is the norm in a routine clinic setting, therefore, it is possible that this may have an implication on the white-coat phenomenon, however standardization was ensured by using an automated, validated BP device and same method of BP measurements.
- Our results were not stratified according to age and gender, it is possible that different age groups and different gender groups may show a difference in BP trends.
- The study was conducted in cardiology clinics at a single center, therefore, generalization may be limited.

## Abbreviations

ABPM: Ambulatory blood pressure monitoring
BP: Blood pressure
COBRA-BPS: (Control of Blood Pressure and Risk Assessment – Bangladesh, Pakistan, Sri Lanka)
DBP: Diastolic blood pressure
PC BP: Post clinic blood pressure
PC-ABP: Post clinic ambulatory blood pressure
SBP: Systolic blood pressure
SD: Standard deviation
